# 2338 Identifying the genetic determinants of human brown adipose tissue

**DOI:** 10.1017/cts.2018.82

**Published:** 2018-11-21

**Authors:** Tobias Becher, Paul Cohen, Andreas Wibmer, Daniel J. Kramer

**Affiliations:** 1 The Rockefeller University; 2 Department of Radiology, Memorial Sloan Kettering Cancer Center; 3 Laboratory of Molecular Metabolism, The Rockefeller University

## Abstract

OBJECTIVES/SPECIFIC AIMS: Brown adipose tissue (BAT) increases energy expenditure by dissipating chemical energy as heat. The combustion of glucose and lipids produces beneficial metabolic effects and renders BAT an attractive target to battle obesity and associated diseases. The majority of adults do not display active BAT on positron emission tomography (PET) without prior cold exposure. Interestingly, a fraction of individuals with BAT positive PET scans exhibits excessive BAT (eBAT) activity, indicating a possible underlying genetic contributor. We aim to identify genetic determinants of BAT activity by studying individuals with eBAT activity using next-generation sequencing. A cellular model will be used to validate variants and perform in-depth pathway analysis. METHODS/STUDY POPULATION: We performed a retrospective review of PET scans over a period of 12 months in patients presenting with suspected or diagnosed cancer (n=20,348). The distribution of BAT positive individuals (n=1251) was used to implement a threshold to define eBAT activity. Samples from prospectively recruited individuals with BAT activity above the threshold will undergo whole exome sequencing. Variants associated with eBAT activity will be engineered into an immortalized BAT cell line using CRISPR to validate results and perform in-depth pathway analysis. RESULTS/ANTICIPATED RESULTS: We expect to identify genetic variants associated with eBAT. Studying the effects of these variants on thermogenesis followed by in-depth pathway analysis in genetically engineered cellular and mouse models may enable us to find new regulators of BAT activity. These findings may eventually contribute to the development of new drugs targeting obesity and its sequelae. DISCUSSION/SIGNIFICANCE OF IMPACT: The contribution of genetic factors to individual BAT activity is currently unknown. Identifying individuals with eBAT on PET scans and studying the underlying genetic determinants may provide the foundation for the discovery of new pathways for BAT activation.

